# THE IMPACT OF ALIVIADO DEMENTIA CARE HOSPICE EDITION TRAINING PROGRAM ON HOSPICE STAFF’S DEMENTIA SYMPTOM KNOWLEDGE

**DOI:** 10.1093/geroni/igz038.3130

**Published:** 2019-11-08

**Authors:** Catherine E Schneider, Alycia A Bristol, Ariel Ford, Shih-Yin Lin, Abraham A Brody

**Affiliations:** 1 New York University, New York, New York, United States; 2 NYU Rory Meyers College of Nursing, New York, New York, United States

## Abstract

A lack of high quality dementia training for healthcare workers is a key barrier to effective care for persons with dementia (PWD), a vulnerable and increasing population across the care continuum. Hospice agencies in particular are underprepared to care for this population, although annually about 17% of hospice patients have a primary diagnosis of dementia and an additional 28% as a comorbidity. Aliviado Dementia Care-Hospice Edition is an interdisciplinary, evidence-based quality improvement program developed to assist hospice interdisciplinary teams in caring for PWD and their caregivers. Interdisciplinary hospice team members in two agencies were enrolled in online training modules, which addressed multiple areas including pain, behavioral and psychological symptoms of dementia (BPSD), and working with caregivers. They were also provided a toolkit to integrate training in daily practice. Changes in knowledge, confidence and attitudes were tested before and after training and paired t-tests were utilized to evaluate the program’s effect. Thirty-five individuals completed the program and pre/post tests. Paired t-tests showed clinically and statistically significant increases in knowledge, attitudes and confidence in five of 10 domains including depression knowledge and confidence and BPSD knowledge, confidence and interventions. The greatest increase was in using BPSD interventions (18.5% increase, p-value: 0.0002), depression confidence (15.9% increase, p-value: 0.006) and BPSD confidence (12.6% increase, p-value: 0.02). Aliviado is an evidence-based, systems-level intervention shown to improve clinical knowledge, attitudes and confidence in treating pain and BPSD in PWD. This training could be used to produce systems-level practice change for hospice interdisciplinary team members serving PWD.

